# Markers of Excellence: Professional Development Opportunities in an Organic Chemistry CURE

**DOI:** 10.1021/acs.jchemed.5c00992

**Published:** 2025-12-26

**Authors:** Evelyn A. Boyd, Clark I. Andersen, Joi P. Walker

**Affiliations:** † Department of Chemistry and Biochemistry, 8083 University of Mississippi, 322 Coulter Hall, University, Mississippi 38655, United States; ‡ Department Chemistry, 3627 East Carolina University, 300 Science & Technology Building, Greenville, North Carolina 27858, United States

**Keywords:** Second-Year Undergraduate, Chemical Education Research, Organic Chemistry, Inquiry-Based/Discovery Learning, Undergraduate Research

## Abstract

The American Chemical Society has developed professional development competencies to guide the integration of professional skills into chemistry curricula. This study examines how these competencies were demonstrated in an organic chemistry course-based undergraduate research experience (CURE) with a unique emphasis on team science. This CURE exhibited each of the five professional development competencies at the highest level, providing a model for how instructors can enhance the professional development impact of their courses. By embedding explicit team science training within a CURE, students gained stronger preparation for collaborative, real-world scientific work than traditional laboratory courses typically provide.

## Graphical abstract

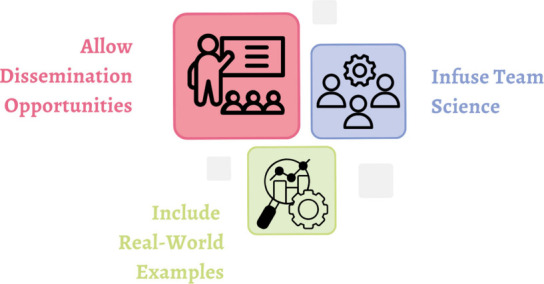

Chemistry departments are in crisis as fewer and fewer students are declaring and graduating in the majors.^1^ Several factors contribute to this trend, including a lack of access to well-trained chemistry teachers in K-12 settings,^2,3^ concerns about family views of chemistry,^3–5^ poor grades early in the major course sequence,^3,5^ and limited awareness of career pathways.^2,5–7^ Documented methods successful in recruiting and retaining students include making the real-world relevance and professional development opportunities available in the major more apparent to interested students.^8^ Strengthening student professional development skills is also aligned with recent American Chemical Society (ACS) adaptations to programmatic guidelines.^9^

Course-based undergraduate research experiences (CUREs) are well-documented in the literature for their positive impact on student outcomes, including enhanced professional skills.^10–12^ CUREs differ from traditional laboratory courses in that they involve students in authentic research, foster critical thinking, and promote skill development relevant to scientific careers. Corwin et al.^13^ described the importance of implementing science practices, activities used by scientists to acquire new knowledge and solve problems, into CUREs. As a continuation, Buchanan and Fisher^14^ identified specific science practices vital to CUREs:

- students describing hypotheses
- designing methodology
- reviewing primary literature
- disseminating the results

However, Watts and Rodriguez^15^ found that oftentimes, chemistry CUREs do not complete the final step of this process by having students participate in the dissemination of the work in some manner. This dissemination process is important in cementing the outcomes of the CUREs for the students and is perhaps one of the more important pieces for contributing to the professional development outcomes of the CURE.

Another essential skill for professional scientists is collaborating effectively in research teams.^16^ The Science of Team Science (SciTS) which describes principles that enhance collaboration (i.e., effective communication, conflict resolution skills, and project planning) are key for productive teamwork among scientists.^17^ Unfortunately, few programs explicitly teach team science skills to undergraduate students.^18^ Embedding team science training into undergraduate courses could be a particularly powerful way to prepare students for scientific workplaces. Structured team training within a CURE provides students with opportunities to develop both research skills and team science competencies.^19–21^

In 2023, the ACS Committee on Professional Training published revised guidelines for bachelor’s degree programs. These guidelines are intended to direct chemistry departments in assessing their course of study and explaining the requirements for ACS certification of bachelor’s degrees. As part of these guidelines, professional skills and competencies are described in a rubric format of five competencies:

- communication skills
- information retrieval, evaluation, and management
- teamwork and collaboration
- professional conduct of scientists
- systems thinking

These five competencies may each be displayed at one of three levels, critical requirements, normal expectations, and markers of excellence.^9^ Professional skills and competencies were developed with the intention of infusion throughout the degree program and do not need to be demonstrated in any one course; however, this paper utilizes the competencies as a metric for determining student described professional development and the situated cognition that takes place within a CURE with an emphasis on SciTS. A previous manuscript in this journal described the original implementation of this CURE.^22^ The present study builds on that work by applying a competency framework to analyze data from a later iteration of the course. This updated version incorporates explicit team-science elements and examines specific professional development outcomes not previously reported. In addition, we provide recommendations for integrating professional development opportunities into chemistry coursework across diverse instructional contexts.

## Theoretical Framework

This study utilizes situated cognition as a theoretical basis. Situated cognition describes how context influences learning.^23^ This theory is often used in studies on CUREs as it allows researchers to differentiate components of the CURE, such as the use of scientific practices, teamwork components, discovery, and relevance, from traditional laboratories.^10,24^ In the context of this CURE, situated cognition allows students to experience professional development opportunities and work through the uncertainty of real research problems with their teams as opposed to following stringent procedures in a traditional lab. Much like language is learned through immersion in its use, scientific culture and practice are learned through active participation in the community. By being embedded in the culture of scientific inquiry, students begin to adopt the norms, values, and practices of the broader scientific community.^16,23,25,26^

## Research Question

The research question of this study is, what are the student-described professional gains after participating in a two-semester organic chemistry CURE?

## Methods

### Organic Chemistry CURE

This work was conducted at a university in the mid-Atlantic United States. At the time of this study, the university was classified by the Carnegie classification system as a doctorate-granting institution with high research activity.^27^ The Chemistry department has developed an Organic CURE sequence, which includes SciTS principles alongside organic chemistry learning outcomes.^22,28^ Students participating in the Organic Chemistry I CURE were recruited from the second semester general chemistry lecture courses, and provided with a link to an online form in which they could express interest. Generally, the form generates a pool of 80 to 100 interested students to fill 36 seats. Chemistry majors were admitted to the CUREs first, and the remaining seats were filled by random selection. Despite chemistry majors being admitted first, they did not comprise the majority of course participants, with the following majors being represented:

- Biology
- Biochemistry
- Chemistry
- Child Life
- Exercise Physiology
- Multidisciplinary Studies
- Public Health

Previous publications established that the CURE students were not significantly different from the selection pool based on sex, GPA, and ethnicity.^28^ This study takes place in the second semester of the CURE. The CURE section met twice a week on consecutive days. Each session lasted 90 min, for a total of 3 h per week. The CUREs described in this study were carefully developed to include the five components of CUREs:

- science practices
- discovery
- relevance
- collaboration
- iteration^10^

Students were allowed to self-select their teams, with the expectation the teams would remain consistent throughout both semesters of the CURE. Despite this, two teams changed at the end of the first semester. One student decided to leave the CURE after the first semester and a new student joined the CURE between semesters to bring the number of students to its original amount. CURE SciTS assignments are described in Table 1. For examples of how SciTS assignments were assessed in a different CURE, see Vance-Chalcraft et al.^29^ These course activities were first developed and assigned and later evaluated using the ACS professional competencies.^9^

**Table 1:** CURE Assignments

Assignment	Description
Individual Writing Prompt	An individual writing prompt was completed to promote further investigation into the research topic and engagement with scientific literature. Students were instructed to create a proposal for future work related to their current research project. The proposal needed to include synthetic methods, selection of target compounds, an assessment of the environmental impacts of the proposed synthesis, in addition to a description of a team-based approach that will achieve these research goals.
Notebook Checks	Three notebook checks to ensure proper record keeping took place throughout the semester.
Literature Meetings	Two discussions of instructor approved peer review papers took place within the semester.
Research Plan	Teams designed research plans including a short description of the research project, information about the intended final product, and tasks that needed to be completed along with how these tasks contribute to the end goal of the project. Teams were encouraged to assign tasks to individuals in this plan. These plans were revisited and updated frequently throughout the semester.
Communication Plan	Teams created communication plans following SciTS standards. These plans discussed preferred modes and frequency of communication, conflict resolution strategies, and course or project specific situations. These plans were revisited and updated frequently throughout the semester.
Poster and Presentation	Teams created a poster of their research and future directions and presented them on the last day of class.
Research Paper	Teams submitted a final research paper in sections (Introduction, Experimental, and Results) throughout the second half of the semester. Each student was required to participate as a lead author for at least one of the sections.

### Team Science Training

Graduate student teaching assistants (GTAs) were hired to provide team science training and manage team science elements in the CUREs. The GTAs were required to attend two team science training workshops to familiarize themselves with team science principles and how they would be implemented in the CURE. These workshops were led by the research team, and all GTAs attended and participated. In the first workshop, GTAs were instructed on the relevance of SciTS and how it should be implemented. Team science competencies were modeled by establishing norms to be followed during the workshop, including

- full participation
- listening
- honoring timeframes
- criticizing ideas, not people

The workshop objectives were to discuss the significance of teams in science, to describe factors that make scientific teams effective, and to describe expectations for what the teams needed to do to be successful. Outlines for effective communication and research planning were provided to the GTAs, and they were instructed to require student teams to develop similar plans in the first week of the CURE. The second GTA training took place during the third week of the semester and focused on conflict resolution. The goals for this second training included discussing different types of conflict that can occur in teams, discussing the important benefits of conflict, and practicing how to recognize positive and negative conflict. This training was performed after CURE work had begun in earnest because it was at this point that some GTAs might have begun to see conflict arising among the student teams. Activities in this workshop included brainstorming potential sources of conflict in the lab and participating in case study role plays to practice managing a conflict in a team. The second workshop was designed to solidify the GTA’s understanding that conflict can lead to an increase in work effectiveness if handled properly. Examples of conflict role play activities, research plan guidance, and communication plan guidance have been provided in the Supporting Information (SI 2). Additionally, a guide for implementing team science into your courses is available.^30^

### Data Collection

Thirty-five students were enrolled in the Organic Chemistry II CURE, a total of 12 participants (34%) engaged in one of three focus groups in the final weeks of the spring semester. Most of the focus group participants (*N* = 11, 92%) participated in the CURE for two consecutive semesters and worked with the same team throughout.

The focus group protocol was informed by Wierzchowski and Wink,^24^ who used Situated Cognition^23^ as a framework to assess meaningful learning in a CURE setting. Focus groups were conducted by two researchers familiar with the CURE and focus group methods and had no grading responsibilities for the CURE. They were conducted in person and recorded utilizing Microsoft Teams. One of the researchers attended via Microsoft Teams, allowing for real-time monitoring of recording quality and ensuring all voices were captured. Microsoft Teams also provided a written transcript of the focus group. Students were invited to participate in the focus groups during their course meeting times, and the focus groups took place directly after class times. Each focus group lasted approximately 30 min, with semistructured questions designed to explore how students designed and participated in the research projects and research dissemination contributed to their overall experience in the CURE. The focus group protocol with the questions asked is available in the Supporting Information (SI 1).

### Data Analysis

Transcripts were cleaned and verified for accuracy before being analyzed. An inductive coding approach was initially employed to identify emergent codes from the data. Following this, a deductive thematic analysis was conducted using the ACS Professional Skills and Competencies^9^ as a guide. The ACS Professional Skills and Competencies are divided into five categories:

- communication skills
- information retrieval, evaluation, and management
- teamwork and collaboration
- professional conduct of scientists
- systems thinking^9^

Consensus coding was performed among two researchers for both the coding and thematic analysis and full agreement was obtained. Codes and themes were reviewed iteratively, ensuring alignment with both emergent insights and established professional competencies.

Additionally, the course syllabus was reviewed alongside focus group transcripts to contextualize student responses and verify the frequency and structure of professional development opportunities throughout the course. The course assignments were evaluated for alignment with the ACS Professional Competencies by comparing the data to the requirements described for each level of the rubric, critical requirements, normal expectations, and markers of excellence.^9^ This study was approved exempt from Institutional Review Board (UMCIRB: 20-000808) review by the supporting institution. No unexpected or unusually high safety hazards were encountered during this study.

## Results

The CURE students performed in each of the five ACS professional development competencies at the highest level, “Markers of Excellence”. The competencies, requirements for achieving “Normal Expectations” and “Markers of Excellence” as described in the guidelines,^9^ CURE assignments that align with each competency, and the frequencies that each competency area was coded in the focus group transcripts are summarized in Table 2. While frequencies of codes are presented, the quantity of a code alone is not an effective indicator of its importance in qualitative research. Coding in qualitative analysis aims to capture the depth and meaning of participants’ experiences, providing a richer understanding of the data than frequency counts alone can convey. Focus group quotes and further evidence of how each competency was met at the “Markers of Excellence” level are presented throughout the remainder of the Results section.

**Table 2:** ACS Professional Competencies at a Markers of Excellence Level

Competency	Normal Expectations Requirements^9^	Markers of Excellence Requirements^9^	CURE Activity Alignment	Frequency of Codes
Communication Skills	Communication skills are developed across the curriculum with multiple opportunities for practice and evaluative feedback.	Knowledge of more than one language or an international experience to contribute to student’s ability to communicate with chemists worldwide.	• Individual Writing Prompt	18
		Offering opportunities that go beyond coursework for students to engage with the broader institutional, local, or scientific community.	• Literature Meetings	
			•Communication Plans	
			• Research Paper	
			• Poster and Presentation	
Information Retrieval, Evaluation, and Management	Instruction is provided in data management and archiving, record keeping (electronic and otherwise), and managing citations and related information.	Students demonstrate knowledge of intellectual property issues associated with scientific publications including author’s rights, the use of copyrighted materials in research and instruction, the peer review process, and publication in and access to open-access journals.	• Individual Writing Prompt	3
	Students are trained in strategies for assessing the quality of sources of scientific information.		• Notebook Checks	
			• Literature Meetings	
			• Research Plan	
			• Poster and Presentation	
			• Research Paper	
Teamwork and Collaboration	Approved programs should incorporate effective measures to assess the performance of both team leaders and members across the curriculum.	Provide instruction and assessment of a range of skills including effective coordination, direction, and engagement of team members; experience in persuasion and negotiation to accomplish goals and meet deadlines; and ability to resolve conflicts and critically evaluate team members.	• Notebook Checks	6
		Provide opportunities for students to build strong communication skills and initiative, respect for the views of other team members, and reliability and commitment to the task at hand.	• Literature Meetings	
			• Research Plan	
			•Communication Plan	
			• Poster and Presentation	
			• Research Paper	
Professional Conduct of Students	Successful programs prepare their students to recognize the impact of their work on individuals in society.	Assessment of professional conduct should go beyond evaluating student reports or laboratory notebooks.	• Individual Writing Prompt	8
	Students have opportunities to learn to treat individuals with respect and fairness in all aspects of the scientific process: Establishing collaborations, partnerships, and mentoring relationships.	Programs provide multiple opportunities for students to engage in self-reflection and discussion of the role of sustainability, bias, policy/regulation, professional growth, limitations of knowledge in the practice of science.	• Literature Meetings	
	Designing and conducting research projects.		• Research Plan	
	Writing and reviewing manuscripts and proposals; presenting research findings at conferences, etc.		•Communication Plan	
			• Poster and Presentation	
Systems Thinking	Students should be made aware that solutions to problems in the world around us require decision-making that takes into consideration chemical knowledge as well as social, economic, political, moral, or environmental factors.	Students work through problems that bring in chemical knowledge, as well as social, economic, political, moral, or environmental factors.	• Individual Writing Prompt	4
			• Literature Meetings	
			• Research Plan	

### Communication Skills

“Markers of Excellence” for student communication skills include “knowledge of more than one language or an international experience and offering opportunities that go beyond coursework for students to engage with the broader institutional, local, or scientific community.”^9^ CURE students did not have direct evidence of international experiences; however, they did engage with real literature and their research had the potential to have a global impact. In focus groups, communication skills were mentioned most often in conversations (coded 18 times, Table 2). Participants described opportunities to practice and expand on communication skills through the development and presentation of a final report and poster presentation that took place at the end of both semesters of the CURE. Additionally, students were provided opportunities to communicate extensively with their teams while following their communication plans and in literature meetings. The individual writing prompt contributed to written communication skill development by requiring students to detail how they would communicate in their teams. Students appreciated having multiple forms of communication and in focus groups expressed that working on one assignment helped them better understand and feel more prepared for the other assignments, as exemplified here:

“I just feel like being able to present and then have people ask you questions rather than just having people ask you questions and read it by themselves ... definitely helps you get a better understanding of things. Also, because ... people learn better from being able to teach other people about something that they’re studying.”

Students had multiple opportunities to receive iterative feedback from fellow students, GTAs, and professors. This included teams practicing their presentations with other teams. During the focus groups, one participant shared how viewing another team’s work and the peer review process helped them improve their own work.

“The group we looked at other research and saw what they did well and what they could have improved on and we kind of went from there and filled in the gaps and kind of related it to ours.”

This feedback is imperative to helping the students solidify their learning and understanding of the concepts and improving their presentation skills.^31^ In the focus groups, students mentioned struggles with creating figures and concerns of stage fright during presentations:

“I think the only thing was ... the public speaking because most of us are very ... reserved, we don’t really like talking in front, but I feel like that was able to ... help us get out of our comfort zone and kind of ... realize that ‘oh! it’s not as bad as we think it is!”

However, they also expressed that with practice these things improved and acknowledged the importance of developing these skills in their progression as professionals.

### Information Retrieval, Evaluation, and Management

To achieve a “Markers of Excellence” level in Information Retrieval, Evaluation, and Management students must display “knowledge of intellectual property issues associated with scientific publications including author’s rights, the use of copyrighted materials in research and instruction, the peer review process, and publication in and access to open-access journals”^9^ (Table 2). This skill was mentioned least frequently in the focus groups, however despite the low frequency it was still an important subject for students (frequency 3, Table 2). In the focus groups, students described several interactions with literature to work on and understand their research as exemplified by this conversation between two focus group participants.

“We have to check the credibility [of references] before including them in our paper.”

“Yeah! And [professor] gives us a bunch of files [journal articles] that we can look through to get us started.”

An additional description of student interaction with literature from the focus groups demonstrates how students supplemented course instruction with additional literature to ensure they understood the content.

“[The TA] explained ... [the] technique that we did and if we still didn’t really understand ... how it was done, we would do our own research outside and ... look up what we did, and why that technique is important, and then ... when reading that see the correlation like ... ‘ohh!’”

In addition to these interactions, students participated in two literature discussions per semester in the CURE and the individual writing prompt required students to interact with literature. During the CURE, it was made clear to students that they were working on research projects that would eventually be publishable, and conversations of the peer review process and authorship took place in these contexts.

### Teamwork and Collaboration

To achieve a “Markers of Excellence” level of Teamwork and Collaboration, students must have “experience in leadership development involving providing instruction and assessment of a range of skills including effective coordination, direction, and engagement of team members; experience in persuasion and negotiation to accomplish goals and meet deadlines; and ability to resolve conflicts and critically evaluate team members.”^9^ Additionally, evidence that “the effectiveness of team members is enhanced through opportunities to build strong communication skills and initiative, respect for the views of other team members, and reliability and commitment to the task at hand”^9^ (Table 2).

This course included explicit instruction and training in the SciTS concepts^22^ and all course components were completed as a team with the exception of the individual writing prompt (Table 1). The team science elements included research and communication plans that were updated frequently and contained plans for modes of communication, and conflict resolution alongside plans for completion of research tasks. Teamwork and collaboration were mentioned six times in the focus groups (Table 2). Students described the importance of their teams, and how well they worked together through accounts such as, “*I think in aspects of this semester, we collaborated more. Last semester was very much ‘You had this section. You have this one ... go!’ ... So this semester, we’re actually meeting up discussing things more.*”

### Professional Conduct of Students

Participants demonstrated professional conduct on a “Markers of Excellence” level. To achieve this level, programs must demonstrate student professional conduct by having “assessment of professional conduct beyond evaluating student reports or laboratory notebooks and by providing multiple opportunities for students to engage in self-reflection and discussion of the role of professional growth, and limitations of knowledge in the practice of science”^9^ (Table 2). Professional Conduct of Students was mentioned 8 times in focus groups (Table 2).

Student teams prepared posters for a presentation in front of the class at the end of the semester. Focus group participants reflected on the benefits of these poster presentations and how their TA and instructor offered feedback on both the development of the poster and presentation skills:

“We would go up and give ... a practice presentation the week before our actual presentations ... And so I know that kind of helped ... and then [Professor], and [TA] were like, ‘oh, these are some of the things that you probably missing ... that you should talk more about.’”

Additionally, during the course, students had several opportunities to reflect on their professional growth and the practice of science. This was particularly the case for the students who participated in both semesters of the CURE. The focus groups themselves were an opportunity to reflect on these topics and students shared their reflections accordingly, as one student did here.

“I remember talking a lot about mistakes that were made, but I think even when you’re talking about scientifically doing experiments, mistakes are made. And when you understand those mistakes, you can grow from those mistakes. And from this semester, yeah, we still had some mistakes, but we learned a lot from last semester, so, being able to effectively know when you’ve messed up also shows your knowledge of the material. ...You knew this was supposed to happen, but you did this, and going forward you would change that later.”

In addition to poster creation and presentation, the research and communication plans, literature meetings, and individual writing prompts all contributed to students’ competency ways of conducting themselves professionally.

### Systems Thinking

The students in the CURE exhibited a “Markers of Excellence” level of Systems Thinking competency by affording opportunities for students to “work through problems that bring in chemical knowledge, as well as social, economic, political, moral, or environmental factors”^9^ (Table 2). Systems Thinking skills were mentioned four times in the focus groups (Table 2). The research in this CURE addressed real-world research projects involving the synthesis of Warfarin, an anticoagulation medication. Students demonstrated this proficiency in their focus group reflections through descriptions such as the one here: *“When we’re actually ... physically describing what we did in our work that it kind of ... started to come together, I realized that like ‘ohh! ... All of this has to do with Warfarin!”* Student research plans addressed both short-term and long-term goals of the project, so that teams could see the big picture of their research as they were carrying it out. Literature meetings were discussions on relevant peer-reviewed manuscripts and had the potential to include conversations on the social, economic, political, moral, and environmental factors of the projects discussed. Additionally, the individual writing prompts specifically asked the students to reflect on the environmental impact of their proposed synthesis.

## Discussion

CUREs often do not have opportunities for students to participate in the dissemination of their work.^15^ This final step in the research communication process is an important one to help students tie research ideas together and participate in valuable professional development opportunities.^14,25^ Through an organic chemistry CURE with team science training infused, students had multiple dissemination opportunities and opportunities to develop professional competencies.

To achieve a “Markers of Excellence” level for Communication Skills, courses must show evidence of communication in more than one language or with international experiences and offer opportunities for students to engage with the broader scientific community. This iteration of the CURE did so mostly with conversation and instruction of how research contributes to the broader scientific community. Additionally, some of the CURE teams have presented at a campus-wide research conference with every iteration of the CURE and previous iterations of the CURE worked toward publication of a manuscript of their research results.^32^ Other methods to implement these “Markers of Excellence” could be setting up an international virtual exchange within a course,^33,34^ even if the whole semester is not dedicated to international collaboration, there may be opportunities for courses in multiple countries to video conference with each other or set up global connections at a smaller scale. Additionally, if final presentations are being held, they could be accessible to the broader campus and local community to allow student engagement with those outside of their course.^35^ As campuses have increasing access to video conferencing software, this makes opportunities such as guests visiting and collaborating with courses all the more possible.^36^

The CURE demonstrated information retrieval, evaluation, and management at an exceptional level; however, improvements could be made by including more explicit conversation about journal access concerns and publication in open-access journals and other open educational resources (OERs). University libraries often offer workshops or will send librarians to classes to assist with instruction on different areas of information literacy, including OER use.^37^

Incorporation of systems thinking in this course was subtle throughout the semester. Students worked on and discussed research in a pharmaceutical field and its potential impacts in both social and environmental spheres. A 2019 special edition of this journal addresses systems thinking and contains many suggestions of ways that instructors can incorporate these ideas into their courses to best train our students to consider the broader impact of our work as chemists.^38–40^ For example, the CURE can more clearly implement systems thinking by discussing how this work relates to United Nations Sustainable Development Goal #3: “Good Health and Well-Being”,^41^ or how this work can contribute toward solving broad-scale global challenges.^42–44^ Additionally, courses could implement systems thinking topics by inviting guest speakers to come to class and discuss topics related to sustainability in the pharmaceutical industry. This would allow students to engage with the content and demonstrate potential career paths for them.

The infusion of SciTS training into the CURE was a vital piece of the professional development aspect. This instruction provided an avenue for students to develop teamwork and collaboration skills. The research and communication plans were major pieces of team science training. Not all courses are CUREs, and CUREs require proper support and professional development for the instructors involved.^45^ However, many aspects of this CURE can be, and have been implemented, in more traditional courses, such as students creating communication plans^46^ and carrying out research in teams without the whole course being a CURE.^47^

By ensuring that students were assessed on their professionalism in multiple ways, and multiple settings, this course contributed to their holistic professional skill development. Not only did students participate in communication on multiple fronts, but when they created their communication plans, they were expected to include aspects such as conflict resolution. This creates an environment that more closely resembles real workplaces than many traditional courses, as students are assessed in how they conduct themselves in every aspect of the research process.

## Limitations

As a qualitative study, this study reflects the experiences of the 12 students who participated in the focus groups, and their experiences may not be generalizable to larger populations or other institutions. Focus groups were optional and took place directly after regular class meetings; there was no incentive to participate, thus this study may have been impacted by self-selection bias. This study took place within a small-scale course offered to students who chose to participate, chemistry majors were selected for participation first and the remainder of the seats filled by random selection of interested students. However, the skills developed in this course are beneficial for students regardless of major.

## Conclusions

Students’ needs are constantly shifting, and chemistry programs are increasing their focus on professional development to meet these students’ needs. This manuscript describes modes by which programs can achieve the ACS professional competencies at a markers of excellence level. Programs can better promote the professional development of their students and help train them to be the chemistry leaders of tomorrow that the world needs by

1) embedding SciTS in the undergraduate curriculum,
2) implementing dissemination opportunities such as a final paper and or presentation that allows students to practice science communication skills with iterative feedback and tie together the ideas of the course,
3) considering the infusion of instruction on SciTS to reinforce collaborative ideas, and
4) inclusion of real-world topics and research data wherever possible allowing students exposure to ideas beyond the classroom.

## Supplementary Material









## Notes

SI 1: Focus group protocol (PDF, DOCX)
SI 2: Team science implementation guidance, including conflict role play activities, research plan guidance, and communication plan guidance (PDF, DOCX)
