# Supplementary material for: Markers of Excellence: Professional Development Opportunities in an Organic Chemistry CURE
Source: J Chem Educ. 2025 Dec 26;103(1):131–9. doi: 10.1021/acs.jchemed.5c00992 (PMC12805568; doi:10.1021/acs.jchemed.5c00992)
Supplement: Supplementary file 2 [file ed5c00992_si_002.docx]

# **Markers of Excellence: Professional development opportunities in an organic chemistry CURE – Supplemental**

Evelyn A. Boyd,*^1^ Clark I. Andersen^2^, Joi P. Walker^2^

1. Department of Chemistry and Biochemistry, University of Mississippi, 322 Coulter Hall, University, MS, 38655
2. Department Chemistry, East Carolina University, 300 Science & Technology Building, Greenville, NC, 27858

Focus Group Questions:

How would you describe your understanding of your research topic after creating your scientific poster?

How would you describe your ability to communicate scientific concepts to others when designing your poster?

How would you describe your ability to communicate scientific concepts to others during the scientific poster session?

What is your understanding of how to create a visually appealing and clearly laid out scientific poster?

Please explain the use of sources on your poster.

How did you decide what information to include in your poster?

How did you decide what information should be presented in text and what information should be presented in figures?

What strategies did you use to review your research topic prior to preparing and presenting your poster?  Which strategy/strategies was/were most helpful?

Did you practice giving your presentation before the poster session?  If so, how did you practice?

If you could go back and change the strategies you used to prepare for your poster session, what would you do differently?  What are your reasons for that?

What impact did the feedback you received, including questions during your poster session, have on the understanding of your research topic, your ability to communicate scientific concepts, and your understanding of how to create a visually appealing and clearly laid out poster?

Are there any other aspects of this experience you would like to discuss?
