# Supplementary material for: Markers of Excellence: Professional Development Opportunities in an Organic Chemistry CURE
Source: J Chem Educ. 2025 Dec 26;103(1):131–9. doi: 10.1021/acs.jchemed.5c00992 (PMC12805568; doi:10.1021/acs.jchemed.5c00992)
Supplement: Supplementary file 3 [file ed5c00992_si_003.pdf]

Markers of Excellence: Professional development opportunities in an organic chemistry CURE – Supplemental

Evelyn A. Boyd,*[1] Clark I. Andersen[2], Joi P. Walker[2]

1. Department of Chemistry and Biochemistry, University of Mississippi, 322 Coulter Hall, University, MS, 38655
2. Department Chemistry, East Carolina University, 300 Science & Technology Building, Greenville, NC, 27858

Supplemental Information: Team Science Implementation Examples

Team Science Training Role Play Activities

Instructions: You will need three individuals playing. Two as undergraduate team members and one as the TA.

- One confident, "great" student, made A on last exam, eager to please the instructor. "Jo"
- A second, "mid-level" student, sometimes misses class, did better than expected on most of last exam but missed one important concept. "Alex"
- TA "Sam", First TA assignment. Nervous about this team, has never been a part of a CURE, even as a student. This group has not made as much progress as other groups

Scenario 1: Joe asks Sam after a weekly progress meeting to stay and chat about the project, and of course Sam agrees. Role play Joe and Sam talking (hint: Joe should bring up Alex in the conversation)

- What did Sam do or say that was helpful? What did Joe do that should have been corrected?
- What could Sam have done differently? What could Joe have done differently?

Scenario 2: Sam makes an appointment with Alex. Role play Sam and Alex talking.

- What came up that Sam did well?
- What came up that Sam may do differently?

Summary: You can minimize conflicts through goal setting, planning roles and responsibilities, communication and clear expectations. Some conflicts are unavoidable such as culture, background, personality style etc. In dealing with conflicts, strategize beforehand. Conflicts should not all be avoided but are a chance to strengthen a team's connections

Research Plan Guidance

Team Research Planning

Effective teams make sure that all team members understand the big picture goals of the research before the project begins (i.e., they share the same mental model). Then, regularly during the research, they reflect on their progress so far, adjust what tasks need to be done considering where they are in the research process, and identify how those steps are expected to help them accomplish their project goals. Where possible, teams designate specific individuals to take on particular responsibilities for the project. Although all team members need to be knowledgeable about the project, it is often more efficient to divide tasks among members rather than completing all tasks together. Having designated responsibilities ensures all team members are given the opportunity to contribute (and be held accountable!).

Using a living document, discussion board, or other format designated by your instructor, create a research planning document for your team to which you can add information over the course of the research project. This document can help your team progress more efficiently through the research process and better take advantage of working in a team!

Tips:

- Be specific about the tasks to be completed, who is responsible, by when, and how you will know if you are successful
- Use this plan to keep each other, and yourselves, accountable

At the beginning of the project:

Provide a short description of the research project, along with the intended final product(s). What is the big picture goal of this research?

> ➢ The goal is to make sure that all members of the team share the same idea of the research project and understand what needs to be produced.

Periodically throughout the research process (add to the bottom of the document/discussion board):

- A short reflection of what has been working and what hasn't been working so far with the research project
- A description of any adjustments that need to be made
- A list of the short-term tasks that need to be completed
- A list of who is going to complete each task
- A short description of how these tasks will get your team closer to the big picture research goal

Communication Plan Guidance

Team Communication and Trust

Team Communication and trust is key to forming a high performing and productive team. Please discuss each of the following topics, agree on a plan, and articulate your plan in a written document that specifically addresses each item.

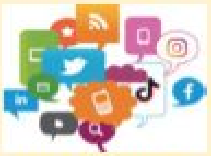

- Mode of communication?
- Frequency
- Time to respond.

Transparency

- Assumptions
- Expectations

Conflict

- Identification
- Resolution Plan

Language

- Speaking
- Listening
- Feedback

"I" represents individual, internal, intentional

- The idea is simple in practice
  - Use statements that refer to your own ideas, feelings, perceptions, motivations
  - Do not use statements that attribute ideas, etc., to others
  - Do not use statements that blame or judge
- This is especially useful during conflict

Elements of a Communication Plan	Low performing Less Productive	High performing More Productive
**General principles for effective, inclusive team communication (~4)**	1 principle	4 or more principles
**Mode of communication (text, slack, teams, email, other)**	Just identified	Multiple modes identified for specific purposes.
**Specify a frequency for communication and time to respond**	Simple response, weekly or during class	Includes quality or detail of communication expectation and specific time to respond.
**How will you handle conflicts that arise between team members?**	Talk to TA or Professor	Multi-stage plan for addressing conflict within the group.
**Course or Project Specific Criteria**	Not considered	Identifies an issue or issues specific to this course and/or project
