# Supplementary material for: Markers of Excellence: Professional Development Opportunities in an Organic Chemistry CURE
Source: J Chem Educ. 2025 Dec 26;103(1):131–9. doi: 10.1021/acs.jchemed.5c00992 (PMC12805568; doi:10.1021/acs.jchemed.5c00992)
Supplement: Supplementary file 4 [file ed5c00992_si_004.docx]

# **Markers of Excellence: Professional development opportunities in an organic chemistry CURE – Supplemental**

Evelyn A. Boyd,*^1^ Clark I. Andersen^2^, Joi P. Walker^2^

1. Department of Chemistry and Biochemistry, University of Mississippi, 322 Coulter Hall, University, MS, 38655
2. Department Chemistry, East Carolina University, 300 Science & Technology Building, Greenville, NC, 27858

**Supplemental Information: Team Science Implementation Examples**

**Team Science Training Role Play Activities**

Instructions: You will need three individuals playing. Two as undergraduate team members and one as the TA.

- One confident, “great” student, made A on last exam, eager to please the instructor.​ “Jo”
- A second, “mid-level” student, sometimes misses class, did better than expected on most of last exam but missed one important concept.​ “Alex”
- TA “Sam”, First TA assignment​. Nervous about this team, has never been a part of a CURE, even as a student​. This group has not made as much progress as other groups

Scenario 1: Joe asks Sam after a weekly progress meeting to stay and chat about the project, and of course Sam agrees​. Role play Joe and Sam talking (hint: Joe should bring up Alex in the conversation)​

- What did Sam do or say that was helpful? What did Joe do that should have been corrected?​
- What could Sam have done differently? What could Joe have done differently?

Scenario 2: Sam makes an appointment with Alex. Role play Sam and Alex talking.

- What came up that Sam did well?​
- What came up that Sam may do differently?

Summary: You can minimize conflicts through goal setting, planning roles and responsibilities, communication and clear expectations​. Some conflicts are unavoidable such as culture, background, personality style etc.​ In dealing with conflicts, strategize beforehand​. Conflicts should not all be avoided but are a chance to strengthen a team’s connections


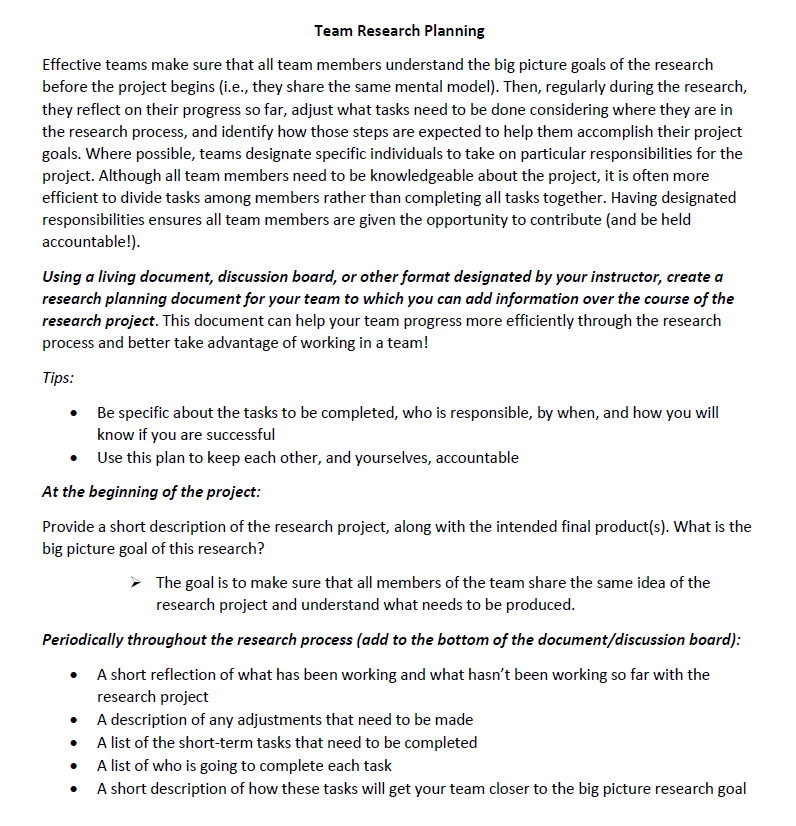


Research Plan Guidance


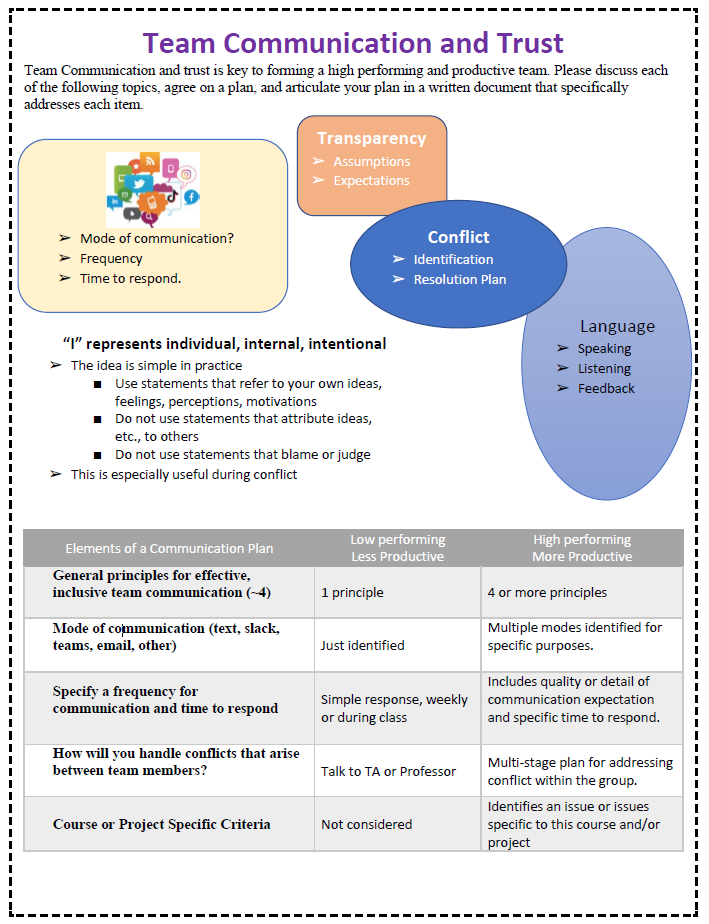


Communication Plan Guidance
